# Complement Drives Chronic Inflammation and Progressive Hydrocephalus in Murine Neonatal Germinal Matrix Hemorrhage

**DOI:** 10.3390/ijms241210171

**Published:** 2023-06-15

**Authors:** Mohammed Alshareef, Devin Hatchell, Tyler Vasas, Khalil Mallah, Aakash Shingala, Jonathan Cutrone, Ali Alawieh, Chunfang Guo, Stephen Tomlinson, Ramin Eskandari

**Affiliations:** 1 Department of Neurological Surgery, Children’s Hospital Colorado, University of Colorado School of Medicine, Aurora, CO 80045, USA; mohammed.alshareef@childrenscolorado.org; 2 Department of Microbiology and Immunology, Medical University of South Carolina, Charleston, SC 29425, USA; hatcheld@musc.edu (D.H.); mallah@musc.edu (K.M.); guoc@musc.edu (C.G.); 3 College of Medicine, Medical University of South Carolina, Charleston, SC 29425, USA; vasasj@musc.edu (T.V.); shingala@musc.edu (A.S.); 4 Department of Family Medicine, AnMed Health Medical Center, Anderson, SC 29621, USA; jonathan.cutrone@anmedhealth.org; 5 Department of Neurological Surgery, Emory University School of Medicine, Atlanta, GA 30322, USA; ali.mostafa.alawieh@emory.edu; 6 Ralph Johnson VA Medical Center, Charleston, SC 29401, USA; 7 Department of Neurological Surgery, Medical University of South Carolina, Charleston, SC 29425, USA

**Keywords:** germinal matrix hemorrhage, intraventricular hemorrhage, neuroinflammation, hydrocephalus, periventricular leukomalacia, complement

## Abstract

Germinal matrix hemorrhage (GMH) is a pathology that occurs in infancy, with often devastating long-term consequences. Posthemorrhagic hydrocephalus (PHH) can develop acutely, while periventricular leukomalacia (PVL) is a chronic sequala. There are no pharmacological therapies to treat PHH and PVL. We investigated different aspects of the complement pathway in acute and chronic outcomes after murine neonatal GMH induced at postnatal day 4 (P4). Following GMH-induction, the cytolytic complement membrane attack complex (MAC) colocalized with infiltrating red blood cells (RBCs) acutely but not in animals treated with the complement inhibitor CR2-Crry. Acute MAC deposition on RBCs was associated with heme oxygenase-1 expression and heme and iron deposition, which was reduced with CR2-Crry treatment. Complement inhibition also reduced hydrocephalus and improved survival. Following GMH, there were structural alterations in specific brain regions linked to motor and cognitive functions, and these changes were ameliorated by CR2-Crry, as measured at various timepoints through P90. Astrocytosis was reduced in CR2-Crry-treated animals at chronic, but not acute, timepoints. At P90, myelin basic protein and LAMP-1 colocalized, indicating chronic ongoing phagocytosis of white matter, which was reduced by CR2-Crry treatment. Data indicate acute MAC-mediated iron-related toxicity and inflammation exacerbated the chronic effects of GMH.

## 1. Introduction

Germinal matrix hemorrhage (GMH) is a hemorrhagic pathology that occurs in premature or low-birth-weight infants and can lead to intraventricular hemorrhage (IVH) [1]. GMH-IVH results in long-term neurologic deficits, posthemorrhagic hydrocephalus (PHH), and periventricular leukomalacia (PVL) [2,3]. PHH and PVL are closely associated with neurodevelopmental delay and cerebral palsy, with 30–42% of infants developing severe disabilities [4,5]. High-grade GMH-IVH results in 90% morbidity and mortality within two years [5]. Surgical treatment of PHH through cerebrospinal fluid (CSF) shunting is the only current therapy to mitigate symptoms, and these procedures carry significant complication risks, including mechanical obstruction and infection [6]. It is well known that infiltrating blood products resulting from GMH are deleterious to the ventricular system and brain by contributing to the development of PHH [7]. Consequently, multiple clinical studies have aimed to reduce the blood-product burden in the brain and ventricular system. The DRIFT trial (drainage, irrigation, and fibrinolytic therapy) was a study evaluating the temporary placement of a ventricular catheter for irrigation and removal of blood products, together with the use of fibrinolytic therapy [8]. Although some benefit was noted, the study was stopped early due to high rates of rehemorrhage in the treatment group [9]. Neuroendoscopic lavage is another technique that has been proposed as a surgical tool for blood-product clearance, with initially lower rates of hydrocephalus in treated patients [10]. There are no approved pharmacological treatments for GMH.

Although there is a causal link between GMH-related blood products and the development of PHH, the underlying mechanism(s) resulting in the development of PHH and PVL remains poorly understood [11]. GMH causes injury via an initial primary mechanism defined by mechanical disruption of blood vessels within the subventricular zone that results in direct damage to brain tissue. The primary injury is followed by a secondary injury mechanism that begins 12–24 h after GMH and is accompanied by the evolution and perpetuation of a neuroinflammatory response that leads to PHH and PVL [12]. Multiple studies have investigated the role of different blood components in mediating post-GMH sequelae [7,13,14]. These studies have generally concluded that red blood cells (RBCs) play a key role and that their breakdown contributes to a disproportionate inflammatory response, likely due to heme and iron release [15,16,17,18,19]. Magnetic resonance imaging (MRI) has been used to evaluate the extent of injury from GMH, which together with other types of analysis, has identified hemoglobin breakdown products within the brain and ventricular walls that can persist for months [20,21]. Iron accumulation subsequently develops along the ventricular walls, causing persistent and extensive damage [22]. Blood products in the post-GMH brain are thought to occur via either RBC breakdown (autolysis) or as a result of immune-mediated phagocytosis [23,24,25]. In the autolysis pathway, RBCs can be lysed by the complement membrane attack complex (MAC), the terminal activation product of the complement pathway, which results in an uncontrolled release of hemoglobin (Hb) [26]. The primary clearance mechanism for free Hb is the CD163–haptoglobin–hemoglobin system [22]. However, operative levels of this system are very low in the brain compared to the periphery [27]. In addition, haptoglobin levels are significantly lower in preterm infants, making Hb clearance in this population even more challenging [28]. Free Hb is further oxidized into met-Hemoglobin (metHb) which has been correlated with an increase in activation of proinflammatory mediators such as TNFα and TLR-4 [22]. In a therapeutic paradigm using the complement inhibitor CR2-Crry, we previously demonstrated that complement activation contributes to an acute/subacute proinflammatory response that leads to PHH following GMH [29]. CR2-Crry is a previously characterized inhibitor that binds at sites of C3 activation (C3d deposition) and inhibits the central C3 activation step of all complement pathways [30]. We have also previously shown that CR2-Crry is neuroprotective in models of adult stroke and traumatic brain injury [31,32]. In this study, we investigate the role of complement in RBC breakdown and clearance and how this relates to chronic pathological outcomes after GMH.

## 2. Results

### 2.1. Germinal Matrix Hemorrhage Results in Complement-Dependent Heme Release and Iron-Dependent Inflammation

**Figure 1:**
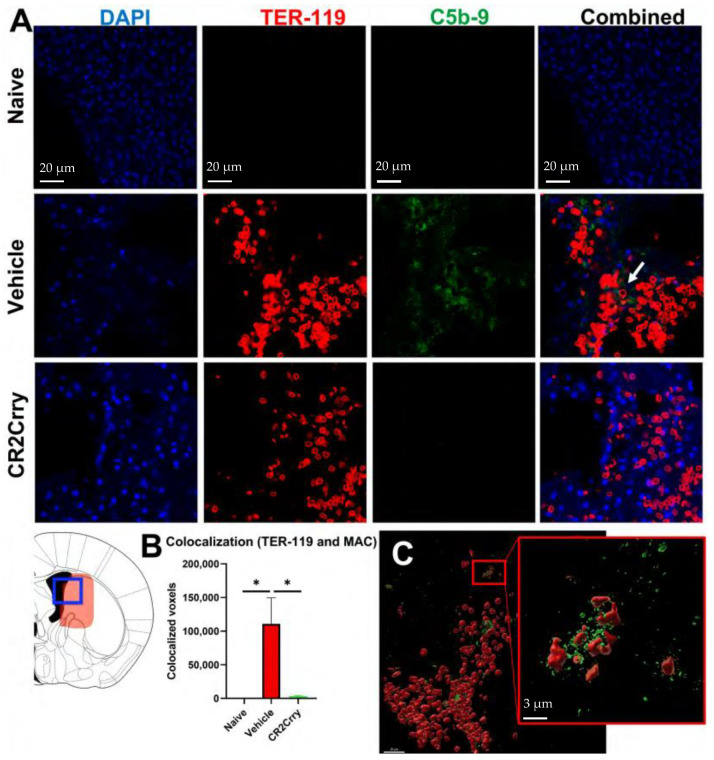
Following GMH, CR2-Crry treatment decreases C5b-9 (MAC) and TER-119 (RBC) colocalization in the injury region acutely at P7. (**A**) Representative IF images for naïve mice, and vehicle-treated or CR2-Crry-treated GMH mice, showing DAPI (blue), TER-119 (red), and C5b-9 (green) and merged TER-119/C5b9 merged images, with increased colocalization within the vehicle image (white arrow). (**B**) Quantification of voxel-based C5b-9 and TER-119 colocalization. One-way ANOVA with Bonferroni’s correction for multiple comparisons. * *p* < 0.05. n = 5 for naïve, n = 9 for vehicle, and n = 9 for CR2-Crry. Error bars = mean ± SEM. (**C**) Representative 3D reconstructed image of a brain section from a vehicle-treated GMH mouse, demonstrating surface coverage of RBCs (red) with C5b-9 (green).

**Figure 2:**
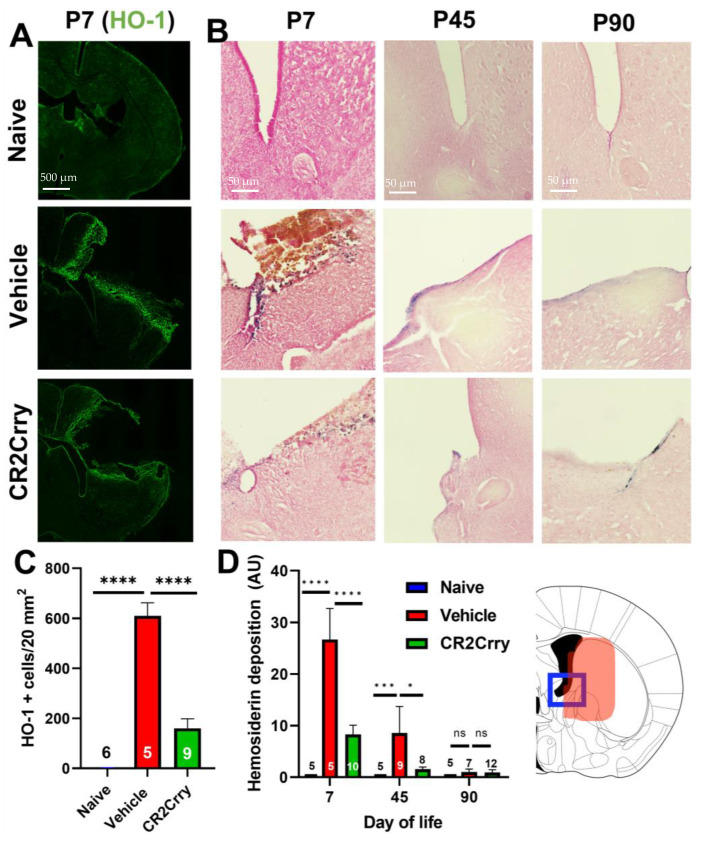
Following GMH, CR2-Crry treatment reduces heme oxygenase-1 expression acutely (P7) and reduces the deposition of extracellular heme at both acute and chronic timepoints. (**A**) Representative IF images of HO-1 staining in brains at P7. (**B**) Representative images of Perls Prussian staining of brains at P7, P45, and P90 demonstrating ventricular heme deposition. (**C**) Quantification of HO-1 staining at P7. One-way ANOVA with Bonferroni’s correction for multiple comparisons. **** *p* < 0.0001. Error bars = mean ± SEM. n = 6 for naïve, n = 5 for vehicle, and n = 9 for CR2-Crry. (**D**) Quantification of Perls Prussian staining in periventricular region of brains at P7, P45, and P90. Blue frame represents the brain region that was imaged corresponding to Panel B and the area quantified in Panel D. One-way ANOVA with Bonferroni’s correction for multiple comparisons within each timepoint. * *p* < 0.05, *** *p* < 0.001, **** *p* < 0.0001. Error bars = mean ± SEM. For P7: n = 5 for naïve, n = 5 for vehicle, n = 10 for CR2-Crry. For P45: n = 5 for naïve, n = 9 for vehicle, n = 8 for CR2-Crry. For P90: n = 5 for naïve, n = 7 for vehicle, n = 12 for CR2-Crry.

We first investigated the role of complement in RBC breakdown acutely after GMH. In vehicle-treated GMH mice at P7 (3 days postinjury), the timepoint representing an early post-hemorrhagic state, MAC (C5b-9) deposition was found to colocalize with RBCs (TER-119) (Figure 1). Little or no MAC was detected in naïve mice or GMH mice treated with CR2-Crry (Figure 1A,B). A representative image of colocalized MAC and TER-119 is shown in Figure 1C. To note, TER-119 staining in the CR2-Crry group is weaker than in the vehicle group. This may be due to reduced MAC-mediated lysis leading to increased RBC phagocytosis and removal. The tissue surrounding the hemorrhage in vehicle-treated GMH mice at P7 contained an increase in heme oxygenase 1 (HO-1)-positive cells, indicating increased heme-related cellular stress (Figure 2A,C). Perls Prussian blue staining also revealed acute heme and iron deposition (hemosiderin) along the edges of the injury site in GMH mice at P7, which was significantly reduced by CR2-Crry treatment (Figure 2B,D). By P45, iron deposition was significantly reduced compared to P7 in vehicle-treated GMH mice but remained significantly higher than in CR2-Crry-treated mice. At P90, there was no evidence of iron deposits in any of the groups.

### 2.2. Complement Inhibition Reduces Chronic Progressive Hydrocephalus and Improves Survival in Chronic GMH Model

**Figure 3:**
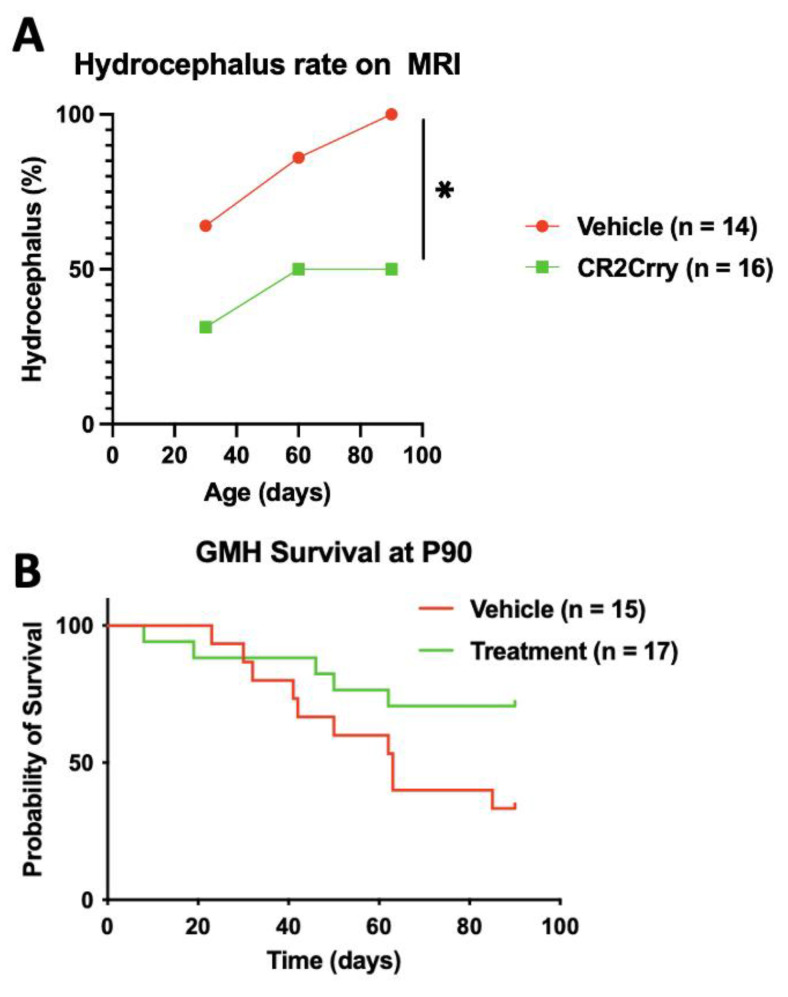
Following GMH, CR2-Crry treatment improves survival and reduces the progressive hydrocephalus rate measured through P90. (**A**) Hydrocephalus (Scale 5 injury) was determined by MRI at P30, P60, and P90; n = 14 for the vehicle and n = 16 for CR2-Crry. The percentage refers to the total number of animals in each group. (**B**) Survival as assessed over 90 days period. Animals that died within the first 24 h of GMH injury were excluded from the analysis. Survival rates were 70% in the CR2-Crry group compared to 33% in the vehicle (*p* < 0.05). Log-rank (Mantel–Cox) test. * *p* < 0.05. n = 15 for vehicle and n = 17 for CR2-Crry.

**Figure 4:**
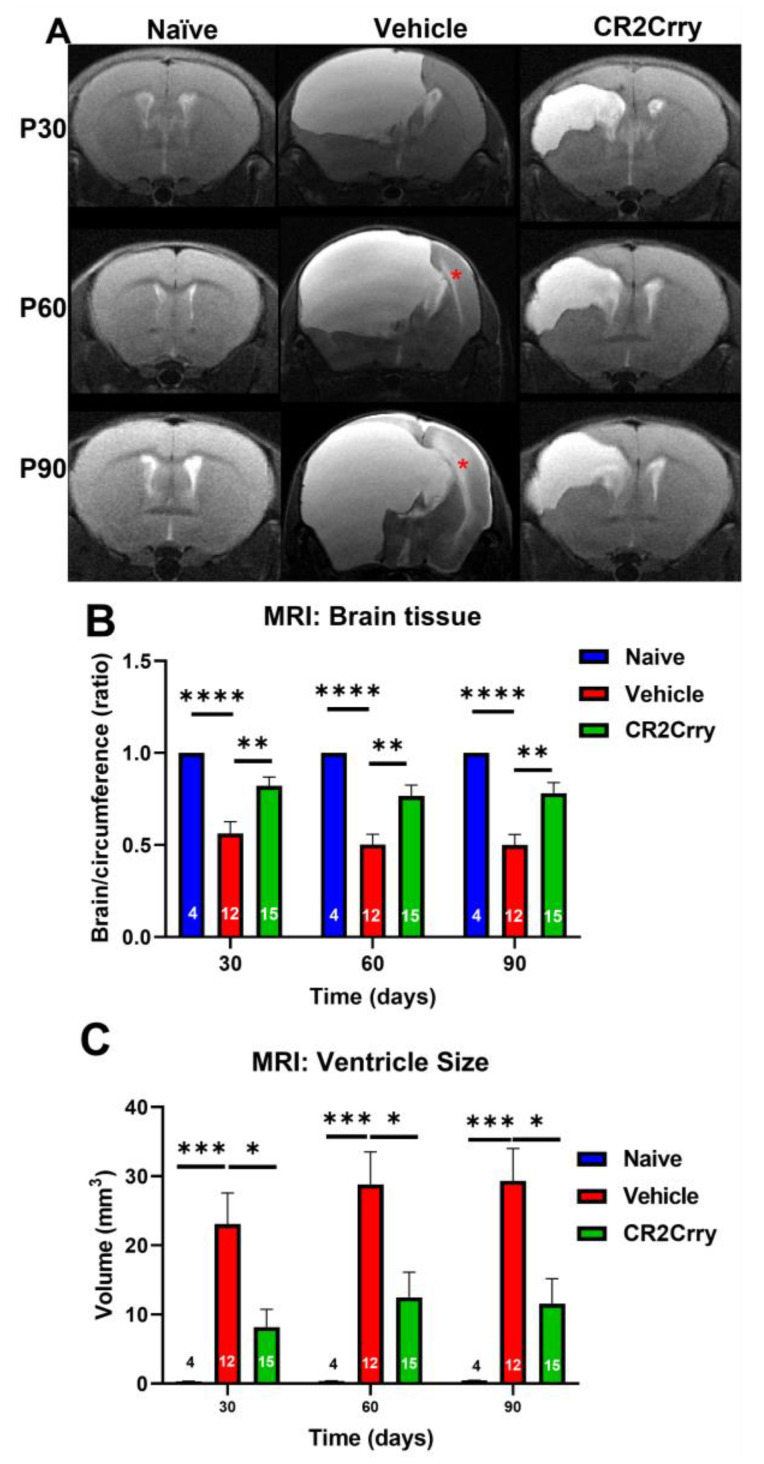
MRI at different timepoints after GMH. CR2-Crry treatment reduces the severity of hydrocephalus chronically after GMH. (**A**) Representative MR mid-ventricle coronal T2-sequence images of naïve, vehicle, and CR2-Crry-treated animals at P30, P60, and P90. The red asterisk represents trans-ependymal flow. (**B**) The ratio between brain area and total cranial area (circumference). The ratio was decreased in vehicle-treated animals compared to CR2-Crry-treated animals. ** *p* < 0.01, **** *p* < 0.0001. Error bars = mean ± SEM. n = 4 for naïve, n = 12 for vehicle, n = 15 for CR2-Crry. (**C**) Ventricular size. Qualitatively, the severity of hydrocephalus and effacement of surrounding structures was evident in vehicle-treated animals more often than in CR2-Crry-treated animals. There was increased transependymal flow over time (seen in the vehicle images), demonstrated by increased brightness surrounding the ventricular system. * *p* < 0.05, *** *p* < 0.001. Error bars = mean ± SEM. n = 4 for naïve, n = 12 for vehicle, n = 15 for CR2-Crry.

**Figure 5:**
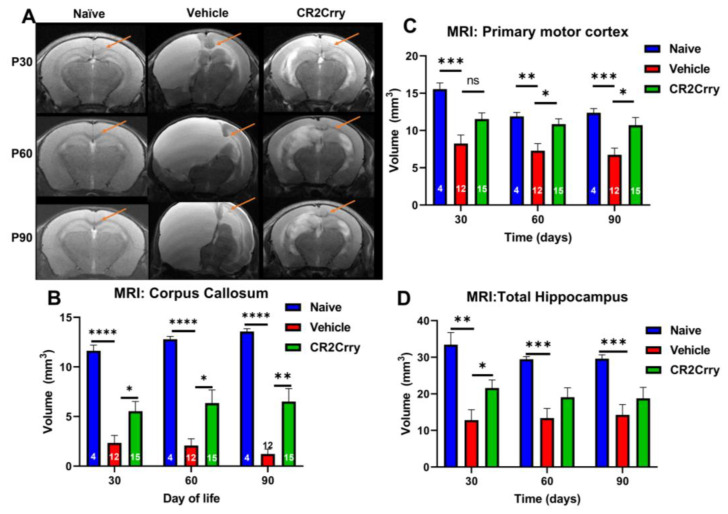
Following GMH, CR2-Crry treatment reduces damage to structures implicated in the development of PVL. (**A**) Representative MRI coronal T2-sequence images of the corpus callosum (arrow), motor area, and hippocampi for naïve, vehicle, and CR2-Crry-treated animals at P30, P60, and P90. (**B**) Corpus callosum volume becomes significantly smaller in vehicle-treated animals compared to naïve and CR2-Crry-treated animals. At P60 and P90, the corpus callosum in the vehicle animals becomes bright because of increased pressure and transependymal flow. * *p* < 0.05, ** *p* < 0.01, **** *p* < 0.0001. Error bars = mean ± SEM. n = 4 for naïve, n = 12 for vehicle, n = 15 for CR2-Crry. (**C**) Motor cortex volume. Identified using an atlas with measurements taken at P30, 60, and 90. * *p* < 0.05, ** *p* < 0.01, *** *p* < 0.001. Error bars = mean ± SEM. n = 4 for naïve, n = 12 for vehicle, n = 15 for CR2-Crry. (**D**) Hippocampus volume. * *p* < 0.05, ** *p* < 0.01, *** *p* < 0.001. Error bars = mean ± SEM. n = 4 for naïve, n = 12 for vehicle, n = 15 for CR2-Crry. ns = not significant.

Magnetic resonance imaging (MRI) was performed at P30, P60, and P90 to evaluate the evolution of hydrocephalus over time. At P30, the rate of hydrocephalus was 64% and 31% in the vehicle and CR2-Crry-treated groups, respectively (Figure 3A). At P90, 100% of vehicle-treated animals had progressed to hydrocephalus, whereas the hydrocephalus rate plateaued at 50% for CR2-Crry-treated animals (Figure 3A). At P90, the survival rate was 70% for CR2-Crry-treated GMH mice, but only 33% for vehicle-treated GMH mice (Figure 3B). Animals that died prior to the first MRI were excluded from hydrocephalus analysis. Animals that were diagnosed with hydrocephalus at the P30 MRI and subsequently died prior to the P60 or P90 MRI were included in the overall hydrocephalus groups at P60 and P90. The assumption was made that hydrocephalus would not reverse over time. Of the surviving animals, none had a reversal of hydrocephalus once developed. MRI analysis was also used to quantify the ventricular volume and remaining brain tissue (Figure 4A). Quantitative analysis of MRI images shows a significantly higher brain-tissue-to-head circumference ratio in naïve and CR2-Crry groups compared to vehicle-treated mice at all three timepoints (Figure 4B). The ventricular size was significantly larger in the vehicle group compared to the naïve and CR2-Crry groups (Figure 4C). A qualitative examination of images at P30, P60, and P90 show worsening of the pre-existing hydrocephalus in the vehicle group, with evidence of transependymal flow (shown by a red asterisk in Figure 5A) and effacement of the surrounding brain tissue. In contrast, CR2-Crry-treated animals develop a stable injury with the minimal progression of the injury or effacement of the periventricular brain structures (Figure 4A).

### 2.3. Chronic Inflammation Following GMH Leads to Radiographic Findings of Periventricular Leukomalacia

To evaluate the consequences of a neuroinflammatory response following GMH, we examined MRI images at P30, P60, and P90 for structural alterations in brain regions with known links to motor and cognitive functions, specifically the corpus callosum, primary motor cortex, and hippocampus (Figure 5A). Corpus callosum volume was significantly larger in naïve and CR2-Crry-treated animals compared to vehicle-treated animals at all timepoints (Figure 5B). The primary motor cortex was measured bilaterally and shown to be significantly larger in the naïve group compared to the vehicle-treated group but not compared to the CR2-Crry group (Figure 5C). While there was no significant difference between CR2-Crry and vehicle at P30, cortex volume was significantly larger in CR2-Crry-treated animals compared to vehicle-treated animals at P60 and P90 (Figure 5C). Of note, progressive hydrocephalus and continued expansion of the ventricles appear to play a role in the effacement and compression of the corpus callosum and primary motor areas, as shown in representative images (Figure 5A). With regard to the hippocampus, volume was significantly larger in CR2-Crry-treated animals compared to the vehicle-treated at P30 but no difference was detected at P60 or P90 (Figure 5D). Hippocampal volume in naïve animals was significantly larger than vehicle animals for all timepoints.

### 2.4. GMH Injury Leads to Increased Gliosis and Phagocytosis of White Matter Chronically after GMH

**Figure 6:**
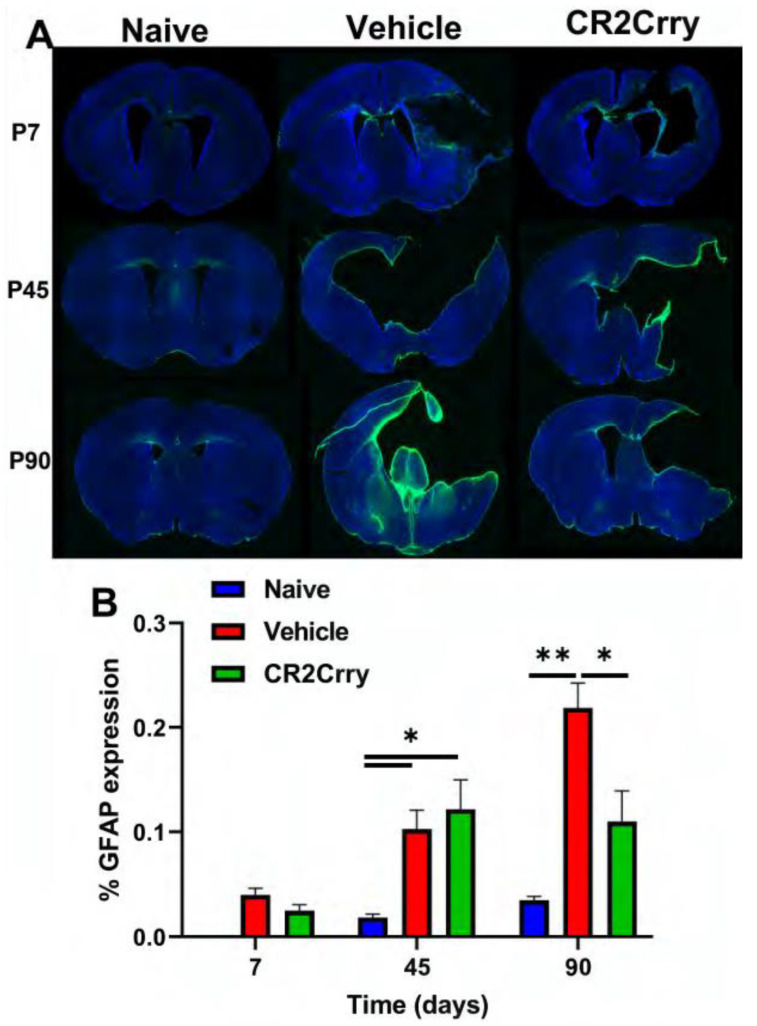
Periventricular astrocytosis is mitigated by CR2-Crry treatment at chronic timepoints after GMH. (**A**) Representative images of GFAP-stained sections from naïve, vehicle-treated, and CR2-Crry-treated animals at P7, P45, and P90. (**B**) Quantification of astrocytosis. * *p* < 0.05, ** *p* < 0.01. Error bars = mean ± SEM. n = 4 for naïve, n = 12 for vehicle, n = 15 for CR2-Crry.

The histologic findings of PVL following GMH include astrogliosis and white-matter tract loss, and we examined animals for astrocytosis at P7, P45, and P90 (Figure 6A). To note, MRI timepoints were at P30, P60, and P90 to allow for serial imaging, while histologic evaluation timepoints allowed examination of tissue at acute, intermediate, and termination timepoints. There was minimal astrocytic accumulation in brains from any group acutely (P7) following injury (Figure 6B). At P45, however, there was a similar and significant increase in periventricular astrocytosis in both vehicle and CR2-Crry groups compared to the naïve group. Astrocytosis plateaued at the P45 level in the CR2-Crry-treated group but increased significantly between P45–P90 in vehicle-treated animals (Figure 6B).

**Figure 7:**
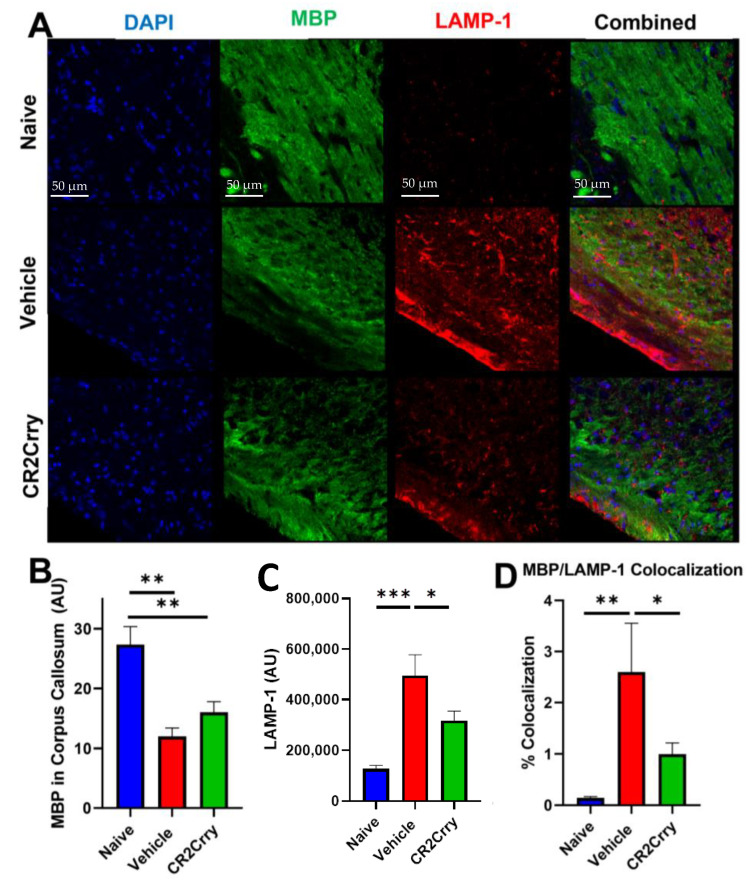
Following GMH, CR2-Crry treatment protects white-matter tracts and reduces white-matter phagocytosis. (**A**) Representative images of DAPI, MBP, and LAMP-1-stained corpus callosum sections at P90. (**B**) Quantification of the overall white-matter area measured by MBP staining in the corpus callosum. ** *p* < 0.01. Error bars = mean ± SEM. n = 4 for naïve, n = 12 for vehicle, n = 15 for CR2-Crry. (**C**) Quantification of LAMP-1 expression in the corpus callosum * *p* < 0.05. *** *p* < 0.001. Error bars = mean ± SEM. n = 4 for naïve, n = 12 for vehicle, n = 15 for CR2-Crry. (**D**) Quantification of MBP and LAMP-1 colocalization. * *p* < 0.05, ** *p* < 0.01. Error bars = mean ± SEM. n = 4 for naïve, n = 12 for vehicle, n = 15 for CR2-Crry.

To evaluate white-matter degradation and phagocytosis, we stained for myelin basic protein (MBP) and the lysosomal marker LAMP-1 at P90 (Figure 7A). There was a reduction in MBP in the corpus callosum in both CR2-Crry- and vehicle-treated GMH animals as compared to naïve animals (Figure 7B). However, the opposite was true for LAMP-1 expression, which was higher in the corpus callosum white matter in vehicle- and CR2-Crry-treated animals compared to naïve animals, and with LAMP-1 expression significantly lower in CR2-Crry-treated vs. vehicle-treated animals (Figure 7C). Furthermore, the level of colocalization of MBP and LAMP-1 was higher in vehicle-treated animals compared to both naïve and CR2-Crry-treated animals, indicating increased phagocytosis of white matter (MBP) in vehicle-treated mice (Figure 7D).

## 3. Discussion

The current work expands our understanding of how complement contributes to neonatal GMH sequelae. Complement inhibition with CR2-Crry reduces hydrocephalus as well as mitigates PVL at chronic timepoints post-GMH, as measured out to P90. Soon after GMH, hemorrhage results in mechanical pressure that results in cell death, cytotoxic edema, and necrosis of surrounding tissue [12,33]. Following mechanical insult, a neuroinflammatory response ensues, in part due to the breakdown of RBCs and uncontrolled hemoglobin release. Hydrocephalus may develop in the early post-GMH period as a result of mechanical obstruction of the cerebral aqueduct and outflow channels [34]; however, even after clearance of blood products, a high rate of hydrocephalus remains, suggesting the occurrence of a separate inflammation-related process [3]. An early key player in GMH-associated hydrocephalus is generally considered to be RBCs [11]. Following hemorrhage, erythrocytes break down and release iron-rich hemoglobin, and iron overload in the brain is associated with CSF overproduction [13] as well as ventricular-wall damage and glial scarring that reduces CSF absorption, resulting in PHH [3]. RBC clearance from the brain can occur by phagocytosis or extracellular cell lysis and the subsequent removal of cell debris.

Macrophages in the periphery, and microglia in the CNS, play a key role in RBC phagocytosis and the safe clearance of iron-containing hemoglobin [35,36]. This process is facilitated by complement-mediated opsonization [37,38] as well as natural antibody-mediated phagocytosis [39]. When RBCs are not phagocytosed, they undergo autolysis, a process that can be driven by the MAC [40]. This results in an uncontrolled release of heme resulting in iron-induced inflammation. Herein, we show that CR2-Crry treatment results in a significant reduction of MAC-RBC colocalization at 3 days after GMH, which, together with our demonstration of reduced heme release and iron-dependent inflammation, suggests a role for the MAC in promoting the post-GMH inflammation seen at more chronic timepoints. To note, increased MAC deposition around the injury site was associated with increased HO-1 expression. Heme oxygenase-1 is a protein found in the endoplasmic reticulum and is expressed following cellular exposure to free heme; its presence has been associated with worse outcomes in hemorrhagic brain pathologies [41,42]. Additional evidence of complement-dependent heme release and subsequent iron release was provided by Perls staining for free iron and metHb, which was higher in vehicle-treated compared to CR2-Crry-treated animals. This difference remained through P45 in vehicle-treated animals. In human studies, heme deposition detected by MRI has been associated with worse performance scores, cognitive decline, and increased occurrence of PHH [21,43]. Thus, the high level of ventricular heme deposition at P45 in vehicle-treated animals may be related to the continued evolution of hydrocephalus. Of note, by P90, heme deposition was not significantly different between vehicle-treated, CR2-Crry-treated, and naive animals. This is consistent with clinical findings in which hemosiderin has been identified by MRI within the first year of life but with decreasing incidence after 1 year [21].

While present acutely after GMH, RBCs are cleared from the brain and CSF within a few days. Mechanisms of heme-related inflammation include activation of TLR-4 and increased expression of TNFα and various interleukins [22]. Prolonged iron-induced inflammation leads to PHH and PVL. PVL is a diagnosis that includes clinical, MRI, and histological findings [44,45]. Clinical findings include cerebral palsy, spasticity, and neurocognitive delay. Imaging-based findings of PVL include cerebral white-matter loss, thinning of the corpus callosum, loss of cortical motor areas, and hippocampal atrophy [46]. Histologic findings include glial scar formation and persistent white-matter inflammation [46,47]. In this study, MRIs performed at 30-day intervals after GMH revealed significantly reduced brain tissue in vehicle-treated animals compared to naïve and CR2-Crry-treated animals. In addition, ventricular size remained significantly higher in vehicle-treated animals compared to the other two groups. In addition, the corpus callosum volume was smaller in vehicle vs. naïve and CR2-Crry groups. Although measurements of the primary motor cortex did not reveal any differences between vehicle and CR2-Crry-treated animals at P30, differences did become significant at P60 and P90. On the other hand, there was a difference in hippocampal size between vehicle and CR2-Crry-treated animals at P30 but not at P60 and P90. Histological analysis of clinical samples has also shown that hippocampal morphology is affected, together with substantial neuronal loss and gliosis [46]. The reduced hippocampal size associated with prematurity has been connected to significant memory deficits on objective testing [48]. Furthermore, studies have shown diffuse white and gray matter abnormalities in preterm infants, irrespective of size, may be associated with worse cognitive outcomes and with the development of seizure disorders [49,50,51].

Neuropathologic evaluation of PVL has demonstrated a large amount of glial scar formation and continued inflammation [45,52]. In this study, we noted no difference in astrocytic scar formation at P7 or at P45. However, astrocytosis levels plateaued at P45 for CR2-Crry-treated animals but continued to increase through P90 in vehicle-treated animals. While there was a reduction in myelin basic protein within the corpus callosum white-matter tracts at P90 in both vehicle- and CR2-Crry-treated animals compared to naive, the LAMP-1 lysosomal marker was significantly elevated in vehicle-treated animals, with a higher colocalization of MBP and LAMP-1 in the vehicle compared to CR2-Crry-treated animals. We also noted a disorganization of white-matter MBP fibers within vehicle-treated compared to CR2-Crry-treated animals, in which a clumped, spheroidal appearance rather than linear fibers was apparent. This qualitative finding has been reported previously in association with axonal injury [52,53].

In a previous study, we demonstrated that complement inhibition reduced C3-dependent microglial phagocytosis of neurons and reduced inflammation at early timepoints after GMH [29]. Here we have followed outcomes chronically and show a role for the terminal complement activation product, the MAC, in early GMH pathogenesis and which appears to impact chronic pathology. We also demonstrate ongoing phagocytosis of white matter as late as 90 days after injury. Our findings introduce a new exploratory paradigm suggesting different complement activation products display temporal effects after GMH. The current data indicate a role for the MAC acutely in post-GMH PHH via RBC lysis and iron-related toxicity, and that this acute MAC-mediated toxicity and inflammation exacerbates chronic pathology seen after GMH. Complement inhibition at the proximal C3 activation step (as with CR2-Crry) additionally mitigates any complement-dependent effects upstream of MAC formation, and in this context, the current data cannot distinguish how earlier complement activation products may also contribute to chronic outcomes. It is also possible that inhibition of C3 acutely after GMH may interfere with the noninflammatory removal of C3 opsonized RBCs and cellular debris, in which case different strategies of complement inhibition after GMH may be optimal; for example, specific inhibition of the MAC acutely after GMH with inhibition more proximal in the pathway, such as at C3 activation, at later timepoints after GMH. Additional studies with different types of complement inhibitors and with mice deficient in different complement proteins/pathways will be able to address these questions.

## 4. Materials and Methods

**Figure 8:**
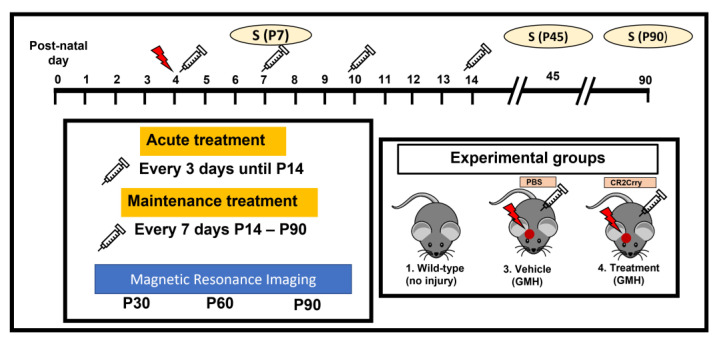
Schematic of study design. Red arrow indicates GMH induction. Syringe indicates PBS or CR2-Crry treatment.

Study design. The study design is summarized in Figure 8. Animal groups in this study were: wildtype naïve (no GMH), vehicle (GMH with intraperitoneal PBS treatment), and CR2-Crry (GMH with intraperitoneal CR2-Crry treatment). Animal breeders and litters were randomly assigned to each of the three groups. A single individual performed all surgeries and treatments, and all GMH injuries were performed by guided injection of collagenase into the subventricular zone (SVZ) on postnatal day 4 (P4), as previously described [29]. Testing and scoring were blinded to group randomization for the duration of the study. Study endpoints were survival, and P7 (3 days postinjury), P45 (41 days postinjury), and P90 (86 days postinjury) for histopathological and immune analysis. For live-animal magnetic resonance imaging (MRI), we performed imaging at P30, 60, and 90 in order to obtain serial imaging for comparison. For the P45 and P90 cohorts, animal genders were identified for animals surviving to P8 (not possible sooner). In the vehicle group, there were 9 females and 6 males. In the CR2-Crry group, there were 9 females and 8 males. In the naïve group, there were 4 males and 4 females.

Animal husbandry and care. The Institutional Animal Care and Use Committee at the Medical University of South Carolina approved all procedures and protocols used in this study. One-month-old wildtype C57BL/J mice (Jackson Laboratory, Bar Harbor, ME, USA) were acclimated for 1 week prior to mating in pairs. Routine cage cleaning was performed, and corn-cob bedding was provided. All mice were exposed to 12 h of light/dark cycles. Continuous access to food and water was provided, and pregnant female mice were additionally given a high-fat diet as recommended by the institutional veterinarian. Cages and litters were checked daily to identify new litters and quantify animal survival. On postnatal day 4 (P4), the male parent was separated from the litter prior to surgery. Following surgery, the pups were placed on a heating pad for 30 min, then reunited with the mother, with a time of 45 min away from the mother. They were monitored for a further 60 min before returning to the mouse housing facility.

Recombinant proteins and treatment paradigm. CR2-Crry was prepared as previously described [54]. Both CR2-Crry and PBS used for intraperitoneal (IP) treatment of animals were endotoxin-free. The complement inhibitory activity of the recombinant protein was verified using a zymosan assay, as previously described [30,54]. CR2-Crry treatment was carried out at 10 mg/kg, a dose previously optimized for adult brain injury [30] and as used by us previously in a GMH model [29]. IP treatments with CR2-Crry or PBS were performed at 1 h after GMH injury, then every 3 days until P13, then every 7 days until either P45 or P90. The frequency of dosing was based on our previous dosing schedule in an adult brain-injury paradigm [55].

Germinal matrix hemorrhage injury model and lesion grading system. The GMH-injury model and grading system were performed as previously described [29]. In short, Clostridium-derived collagenase (Type VII-S collagenase, C2399, Sigma-Aldrich, St. Louis, MO, USA) was injected into the SVZ of mouse pups at P4 to induce direct spontaneous nontraumatic intracerebral hemorrhage in the region of the germinal matrix and SVZ. The injury-grading system is as follows: Grade 0 = No lesion or ventricular enlargement. Grade 1 = Lesion volume < 30% of hemispheric cortical tissue ipsilateral to an injury site without ventricular involvement. Grade 2 = Lesion volume > 30% of hemispheric cortical tissue ipsilateral to the injury site without ventricular involvement. Grade 3 = Lesion extending into the ipsilateral ventricle with no ventricular enlargement. Grade 4 = Lesion extending into the ipsilateral ventricle coupled with unilateral ventriculomegaly. Grade 5 = Lesion extending into both ventricles resulting in global hydrocephalus.

Tissue processing and histologic analyses. Animals were sacrificed at P7, P45, and P90. Following euthanasia, cardiac perfusion was performed with cold PBS followed by 4% Paraformaldehyde (PFA) in PBS. Brains were extracted and fixed in 4% PFA solution overnight at 4 °C, then transferred to 30% sucrose dissolved in 4% PFA in PBS. The brains were embedded in Tissue-Plus Optimal Cutting Temperature (OCT) compound (23-730-571, Fisher Healthcare, Waltham, MA, USA), frozen, and cut in 40 µm coronal sections using a freeze-mount cryostat. Brain tissue was collected in 12-well plates and kept in PBS-filled wells until histologic staining and analysis. For Perls Prussian blue stain, serial brain sections 200 µm apart were mounted on a slide and stained using potassium ferrocyanide as previously described [56]. For ventricular iron deposition measurement, 8 serial-stained brain sections 200 µm apart and 40 µm thick were used to reconstruct the total lesion volume; 4× magnification images of each slice were acquired using a Keyence BZ-X710 microscope (Keyence Co., Itasca, IL, USA). Two independent observers blinded to samples calculated the amount of iron deposition within the histologic samples using NIH ImageJ (FIJI). The average of both observers is reported.

Immunofluorescence staining and imaging. Midhippocampal and midventricular slices were selected using the stereometric measurement from a mouse brain atlas. These slices were stained using standard immunofluorescent (IF) staining as previously described [57]. All imaging and analyses were performed in a blind fashion. High-resolution imaging was performed using a Zeiss LSM 880 confocal microscope (Zeiss, Carl Zeiss Microscopy, LLC, White Plains, NY, USA) at 40× zoom with water–media overlay and with Z-stacking. Images were deconvoluted using the ZEN 2.5 software (Zeiss) and reconstructed in a 3D plane. GFAP was calculated with total signal intensity per total brain area using NIH ImageJ. Negative control images were used to correct for underlying autofluorescence. Myelin basic protein (MBP) was costained with LAMP-1 in brain sections to identify active phagocytosis in P45 and P90 animals. Membrane attack complex (MAC, C5b-9) and red blood cell (TER-119) costaining were performed to identify MAC-involved RBC cytolysis. Colocalization analysis was performed using Imaris (Oxford Instruments, Concord, MA, USA) for 3D image reconstruction and quantification. HO-1+ cells were quantified using NIH ImageJ using a macro that was created for batch processing to maintain uniform analysis. The analysis included background subtraction, conversion to black/white, splitting particles into 20-pixel size, and finally counting the number of individual cells. Primary antibodies used for staining were anti-HO-1 (Abcam, Cambridg, UK. Cat. #: ab11862, 1:200), anti-C5b-9 (Millipore Sigma, St. Louis, MO, USA. Cat. #: 204903-1MG,1:200), anti-TER-119 (Invitrogen, Waltham, MA, USA. Cat. #: 14-5921-82, 1:400), anti-MBP (Abcam, Cat. #: ab40390, 1:150), anti-LAMP-1 (Abcam, Cat. #: ab25245, 1:200), and anti-GFAP (Invitrogen, Cat. #: 13-0300, 1:200). The secondary antibodies utilized were all donkey and included antirabbit Alexa Fluor 488 nm (Invitrogen, Cat. #: A-21206, 1:200), antirat Alexa Fluor 488 nm (Invitrogen, Cat. #: A-21208, 1:200), antirat Alexa Fluor 555 nm (Abcam, Cat. #: ab150154, 1:200), antirabbit Alexa Fluor 555 nm (Invitrogen, Cat. #: A-31572, 1:200), and antigoat Alexa Fluor 647 nm (Invitrogen, Cat. #: A32849, 1:200).

Magnetic Resonance Imaging Technique. MRI was performed at P30, P60, and P85. Imaging acquisition was performed at the Center for Biomedical Imaging/Small Animal Imaging Facility at MUSC. Mice were anesthetized using an isoflurane vaporizer set at the following percentages: 3% for induction and 2% during pilot scanning and data acquisition. After induction, mice were placed in a mouse holder and restrained using a mouse tooth bar (Bruker, Billerica, MA, USA. T10146) and ear bars (Bruker, Billerica, MA, USA. T10147) placed in the auditory canal. Compressed air was used as the carrier gas and delivered at a flow rate of 1 L/min into a nose cone positioned around the tooth bar, where gases mixed with air and passed over the rodent’s nose. All animals were maintained at 37.0 ± 0.2 °C and respiration ranged between 60 and 80 breaths per minute during scanning. The in vivo MRI experiments were all performed on a 7T 30 cm bore scanner (Bruker, BioSpec 70/30 USR) running Paravision version 6.0.1. An 86 mm 1H quadrature volume coil (T128038) was used for signal transmission and an actively decoupled phase array coil (four-channel receiver T11765) was used for signal reception. MRI sequences acquired: (a) T2 weighted anatomy imaging (turboRARE-T2) with the following imaging parameters: TR = 2500 ms, TE = 11 ms, effective TE = 33 ms, RARE Factor = 8, FOV = 20 mm × 20 mm, matrix = 256 × 256, pixel resolution = 0.078 mm × 0.078 mm, slice thickness = 0.7 mm with no gap, slice numbers = 15, number of averages = 2, and scan time = 2 min 40 s; (b) T2 FLAIR (RARE-Inv-Rev) with the following imaging parameters: TR = 10 ms, TE = 12 ms, effective TE = 36 ms, RARE Factor = 8, FOV = 20 mm × 20 mm, matrix = 256 × 256, pixel resolution = 0.078 mm × 0.078 mm, slice thickness = 0.7 mm with no gap, slice numbers = 15, number of averages = 1, scan time = 5 min 20 s; (c) T2star-weighted (FLASH-SWI) with the following imaging parameters: TR = 435.985 ms, TE = 18 ms, Flip angle = 30′, FOV = 20 mm × 20 mm, matrix = 256 × 256, pixel resolution = 0.078 mm × 0.078 mm, slice thickness = 0.7 mm with no gap, slice numbers = 15, number of averages = 3, and scan time = 4 min 11 s. MRI image analysis was performed using NIH ImageJ (FIJI). Measurements were taken by a researcher blinded to the experimental groups. Measurements obtained include ventricle size, brain size, total head circumference, motor area (as identified by a mouse brain atlas), corpus callosum, and hippocampus.

Statistical analysis. Experimental sample sizes were determined by power analysis, and sample size estimation using G*Power 3.1.9.2 tool (Franz Faul, Kiel University, Kiel, Germany). An effect size (d) of 2.0 was determined when comparing GMH mice to naïve and 1.6 when comparing vehicle to CR2-Crry in the treatment group based on our preliminary studies with GMH. An effect size of 1.6 was used in the power analysis for this study. Two-tailed analysis with significance level α = 0.05 was considered with a corrected αc = α/(number of primary comparisons) = 0.05/(2 primary comparisons) = 0.025. The result of the analysis revealed a sample size of 8 evaluable mice per group with an actual computed power of 84%. We expected a 40% potential mortality/exclusion of animals. A final number of 12 animals was required per experimental group to satisfy the necessary minimum. Entire litters were randomized into experimental groups rather than individual pups from a single litter. We performed statistical analysis using GraphPad Prism 8.0 (GraphPad Software, San Diego, CA, USA). Parametric testing was performed unless otherwise specified in the event of a failed Brown–Forsythe test for homogeneity of variance or if normality failed. MRI of hydrocephalus was compared between the groups using the chi-squared test for hydrocephalus rate at each timepoint. Statistical analyses for ventricle, brain tissue, corpus callosum, primary motor area, and hippocampus sizes, as well as IF analyses, were performed using a one-way ANOVA test with Bonferroni’s correction for multiple comparisons. The *p*-values below 0.05 were considered significant. Student’s *t*-test (parametric) was used to compare two groups and was always used as two tailed.

## Data Availability

The datasets generated and/or analyzed for this study are available from the corresponding authors upon reasonable request.

## References

[1] Owens R. (2005). Intraventricular hemorrhage in the premature neonate. Neonatal Netw..

[2] Murphy B.P., Inder T.E., Rooks V., Taylor G.A., Anderson N.J., Mogridge N., Horwood L.J., Volpe J.J. (2002). Posthaemorrhagic ventricular dilatation in the premature infant: Natural history and predictors of outcome. Arch. Dis. Child. Fetal Neonatal. Ed..

[3] Klebe D., McBride D., Krafft P.R., Flores J.J., Tang J., Zhang J.H. (2020). Posthemorrhagic hydrocephalus development after germinal matrix hemorrhage: Established mechanisms and proposed pathways. J. Neurosci. Res..

[4] Bolisetty S., Dhawan A., Abdel-Latif M., Bajuk B., Stack J., Lui K. (2019). New South Wales and Australian Capital Territory Neonatal Intensive Care Units’ Data Collection. Intraventricular Hemorrhage and Neurodevelopmental Outcomes in Extreme Preterm Infants. Pediatrics.

[5] Radic J.A., Vincer M., McNeely P.D. (2015). Outcomes of intraventricular hemorrhage and posthemorrhagic hydrocephalus in a population-based cohort of very preterm infants born to residents of Nova Scotia from 1993 to 2010. J. Neurosurg. Pediatr..

[6] Drake J.M., Kestle J.R., Tuli S. (2000). CSF shunts 50 years on—past, present and future. Childs Nerv. Syst..

[7] Strahle J., Garton H.J., Maher C.O., Muraszko K.M., Keep R.F., Xi G. (2012). Mechanisms of hydrocephalus after neonatal and adult intraventricular hemorrhage. Transl. Stroke Res..

[8] Whitelaw A., Evans D., Carter M., Thoresen M., Wroblewska J., Mandera M., Swietlinski J., Simpson J., Hajivassiliou C., Hunt L.P. (2007). Randomized clinical trial of prevention of hydrocephalus after intraventricular hemorrhage in preterm infants: Brain-washing versus tapping fluid. Pediatrics.

[9] Whitelaw A., Jary S., Kmita G., Wroblewska J., Musialik-Swietlinska E., Mandera M., Hunt L., Carter M., Pople I. (2010). Randomized trial of drainage, irrigation and fibrinolytic therapy for premature infants with posthemorrhagic ventricular dilatation: Developmental outcome at 2 years. Pediatrics.

[10] Schulz M., Buhrer C., Pohl-Schickinger A., Haberl H., Thomale U.W. (2014). Neuroendoscopic lavage for the treatment of intraventricular hemorrhage and hydrocephalus in neonates. J. Neurosurg. Pediatr..

[11] Holste K.G., Xia F., Ye F., Keep R.F., Xi G. (2022). Mechanisms of neuroinflammation in hydrocephalus after intraventricular hemorrhage: A review. Fluids Barriers CNS.

[12] Garton T., Hua Y., Xiang J., Xi G., Keep R.F. (2017). Challenges for intraventricular hemorrhage research and emerging therapeutic targets. Expert Opin. Ther. Targets.

[13] Strahle J.M., Garton T., Bazzi A.A., Kilaru H., Garton H.J., Maher C.O., Muraszko K.M., Keep R.F., Xi G. (2014). Role of hemoglobin and iron in hydrocephalus after neonatal intraventricular hemorrhage. Neurosurgery.

[14] Gao F., Liu F., Chen Z., Hua Y., Keep R.F., Xi G. (2014). Hydrocephalus after intraventricular hemorrhage: The role of thrombin. J. Cereb. Blood Flow Metab..

[15] Mayfrank L., Kissler J., Raoofi R., Delsing P., Weis J., Kuker W., Gilsbach J.M. (1997). Ventricular dilatation in experimental intraventricular hemorrhage in pigs. Characterization of cerebrospinal fluid dynamics and the effects of fibrinolytic treatment. Stroke.

[16] Lodhia K.R., Shakui P., Keep R.F. (2006). Hydrocephalus in a rat model of intraventricular hemorrhage. Acta Neurochir. Suppl..

[17] Chen Z., Gao C., Hua Y., Keep R.F., Muraszko K., Xi G. (2011). Role of iron in brain injury after intraventricular hemorrhage. Stroke.

[18] Gaberel T., Montagne A., Lesept F., Gauberti M., Lemarchand E., Orset C., Goulay R., Bertrand T., Emery E., Vivien D. (2014). Urokinase versus Alteplase for intraventricular hemorrhage fibrinolysis. Neuropharmacology.

[19] Chen Q., Tang J., Tan L., Guo J., Tao Y., Li L., Chen Y., Liu X., Zhang J.H., Chen Z. (2015). Intracerebral Hematoma Contributes to Hydrocephalus After Intraventricular Hemorrhage via Aggravating Iron Accumulation. Stroke.

[20] Meoded A., Poretti A., Northington F.J., Tekes A., Intrapiromkul J., Huisman T.A. (2012). Susceptibility weighted imaging of the neonatal brain. Clin. Radiol..

[21] Yilmaz U., Meyer S., Gortner L., Korner H., Turkyilmaz M., Simgen A., Reith W., Muhl-Benninghaus R. (2016). Superficial Siderosis after Germinal Matrix Hemorrhage. AJNR Am. J. Neuroradiol..

[22] Gram M., Sveinsdottir S., Ruscher K., Hansson S.R., Cinthio M., Akerstrom B., Ley D. (2013). Hemoglobin induces inflammation after preterm intraventricular hemorrhage by methemoglobin formation. J. Neuroinflamm..

[23] Gao C., Du H., Hua Y., Keep R.F., Strahle J., Xi G. (2014). Role of red blood cell lysis and iron in hydrocephalus after intraventricular hemorrhage. J. Cereb. Blood Flow Metab..

[24] Flores J.J., Klebe D., Rolland W.B., Lekic T., Krafft P.R., Zhang J.H. (2016). PPARgamma-induced upregulation of CD36 enhances hematoma resolution and attenuates long-term neurological deficits after germinal matrix hemorrhage in neonatal rats. Neurobiol. Dis..

[25] Flores J.J., Klebe D., Tang J., Zhang J.H. (2020). A comprehensive review of therapeutic targets that induce microglia/macrophage-mediated hematoma resolution after germinal matrix hemorrhage. J. Neurosci. Res..

[26] Ducruet A.F., Zacharia B.E., Hickman Z.L., Grobelny B.T., Yeh M.L., Sosunov S.A., Connolly E.S. (2009). The complement cascade as a therapeutic target in intracerebral hemorrhage. Exp. Neurol..

[27] Galea J., Cruickshank G., Teeling J.L., Boche D., Garland P., Perry V.H., Galea I. (2012). The intrathecal CD163-haptoglobin-hemoglobin scavenging system in subarachnoid hemorrhage. J. Neurochem..

[28] Chavez-Bueno S., Beasley J.A., Goldbeck J.M., Bright B.C., Morton D.J., Whitby P.W., Stull T.L. (2011). Haptoglobin concentrations in preterm and term newborns. J. Perinatol..

[29] Alshareef M., Mallah K., Vasas T., Alawieh A., Borucki D., Couch C., Cutrone J., Shope C., Eskandari R., Tomlinson S. (2022). A Role of Complement in the Pathogenic Sequelae of Mouse Neonatal Germinal Matrix Hemorrhage. Int. J. Mol. Sci..

[30] Huang Y., Qiao F., Atkinson C., Holers V.M., Tomlinson S. (2008). A novel targeted inhibitor of the alternative pathway of complement and its therapeutic application in ischemia/reperfusion injury. J. Immunol..

[31] Alawieh A., Langley E.F., Weber S., Adkins D., Tomlinson S. (2018). Identifying the Role of Complement in Triggering Neuroinflammation after Traumatic Brain Injury. J. Neurosci..

[32] Alawieh A., Tomlinson S. (2016). Injury site-specific targeting of complement inhibitors for treating stroke. Immunol. Rev..

[33] Luo J., Luo Y., Zeng H., Reis C., Chen S. (2019). Research Advances of Germinal Matrix Hemorrhage: An Update Review. Cell. Mol. Neurobiol..

[34] Christian E.A., Melamed E.F., Peck E., Krieger M.D., McComb J.G. (2016). Surgical management of hydrocephalus secondary to intraventricular hemorrhage in the preterm infant. J. Neurosurg. Pediatr..

[35] Lan X., Han X., Li Q., Yang Q.W., Wang J. (2017). Modulators of microglial activation and polarization after intracerebral haemorrhage. Nat. Rev. Neurol..

[36] Jing C., Bian L., Wang M., Keep R.F., Xi G., Hua Y. (2019). Enhancement of Hematoma Clearance With CD47 Blocking Antibody in Experimental Intracerebral Hemorrhage. Stroke.

[37] Mevorach D., Mascarenhas J.O., Gershov D., Elkon K.B. (1998). Complement-dependent clearance of apoptotic cells by human macrophages. J. Exp. Med..

[38] Lin Z., Schmidt C.Q., Koutsogiannaki S., Ricci P., Risitano A.M., Lambris J.D., Ricklin D. (2015). Complement C3dg-mediated erythrophagocytosis: Implications for paroxysmal nocturnal hemoglobinuria. Blood.

[39] Lutz H.U. (2012). Naturally occurring autoantibodies in mediating clearance of senescent red blood cells. Adv. Exp. Med. Biol..

[40] Cao S., Zheng M., Hua Y., Chen G., Keep R.F., Xi G. (2016). Hematoma Changes During Clot Resolution After Experimental Intracerebral Hemorrhage. Stroke.

[41] Wang K.C., Tang S.C., Lee J.E., Lai D.M., Huang S.J., Hsieh S.T., Jeng J.S., Tu Y.K. (2014). Prognostic value of intrathecal heme oxygenase-1 concentration in patients with Fisher Grade III aneurysmal subarachnoid hemorrhage. J. Neurosurg..

[42] Okubo S., Xi G., Keep R.F., Muraszko K.M., Hua Y. (2013). Cerebral hemorrhage, brain edema, and heme oxygenase-1 expression after experimental traumatic brain injury. Acta Neurochir. Suppl..

[43] Imaizumi T., Chiba M., Honma T., Niwa J. (2003). Detection of hemosiderin deposition by T2*-weighted MRI after subarachnoid hemorrhage. Stroke.

[44] Deng W., Pleasure J., Pleasure D. (2008). Progress in periventricular leukomalacia. Arch. Neurol..

[45] Zaghloul N., Patel H., Ahmed M.N. (2017). A model of Periventricular Leukomalacia (PVL) in neonate mice with histopathological and neurodevelopmental outcomes mimicking human PVL in neonates. PLoS ONE.

[46] Pierson C.R., Folkerth R.D., Billiards S.S., Trachtenberg F.L., Drinkwater M.E., Volpe J.J., Kinney H.C. (2007). Gray matter injury associated with periventricular leukomalacia in the premature infant. Acta Neuropathol..

[47] Kinney H.C., Panigrahy A., Newburger J.W., Jonas R.A., Sleeper L.A. (2005). Hypoxic-ischemic brain injury in infants with congenital heart disease dying after cardiac surgery. Acta Neuropathol..

[48] Isaacs E.B., Lucas A., Chong W.K., Wood S.J., Johnson C.L., Marshall C., Vargha-Khadem F., Gadian D.G. (2000). Hippocampal volume and everyday memory in children of very low birth weight. Pediatr. Res..

[49] Inder T.E., Wells S.J., Mogridge N.B., Spencer C., Volpe J.J. (2003). Defining the nature of the cerebral abnormalities in the premature infant: A qualitative magnetic resonance imaging study. J. Pediatr..

[50] Inder T.E., Warfield S.K., Wang H., Huppi P.S., Volpe J.J. (2005). Abnormal cerebral structure is present at term in premature infants. Pediatrics.

[51] Tosun D., Dabbs K., Caplan R., Siddarth P., Toga A., Seidenberg M., Hermann B. (2011). Deformation-based morphometry of prospective neurodevelopmental changes in new onset paediatric epilepsy. Brain.

[52] Hirayama A., Okoshi Y., Hachiya Y., Ozawa Y., Ito M., Kida Y., Imai Y., Kohsaka S., Takashima S. (2001). Early immunohistochemical detection of axonal damage and glial activation in extremely immature brains with periventricular leukomalacia. Clin. Neuropathol..

[53] Billiards S.S., Haynes R.L., Folkerth R.D., Borenstein N.S., Trachtenberg F.L., Rowitch D.H., Ligon K.L., Volpe J.J., Kinney H.C. (2008). Myelin abnormalities without oligodendrocyte loss in periventricular leukomalacia. Brain Pathol..

[54] Atkinson C., Song H., Lu B., Qiao F., Burns T.A., Holers V.M., Tsokos G.C., Tomlinson S. (2005). Targeted complement inhibition by C3d recognition ameliorates tissue injury without apparent increase in susceptibility to infection. J. Clin. Investig..

[55] Mallah K., Couch C., Alshareef M., Borucki D., Yang X., Alawieh A., Tomlinson S. (2021). Complement mediates neuroinflammation and cognitive decline at extended chronic time points after traumatic brain injury. Acta Neuropathol. Commun..

[56] Van Duijn S., Nabuurs R.J., van Duinen S.G., Natte R. (2013). Comparison of histological techniques to visualize iron in paraffin-embedded brain tissue of patients with Alzheimer’s disease. J. Histochem. Cytochem..

[57] Alawieh A., Langley E.F., Tomlinson S. (2018). Targeted complement inhibition salvages stressed neurons and inhibits neuroinflammation after stroke in mice. Sci. Transl. Med..

